# Cost-effectiveness of switching to S-1 after fluoropyrimidine-induced hand-foot syndrome or cardiovascular toxicity in the treatment of metastatic colorectal cancer

**DOI:** 10.1016/j.esmoop.2026.106304

**Published:** 2026-03-17

**Authors:** R. van Eekelen, C.J.A. Punt, J.J.M. Kwakman, V.M.H. Coupé

**Affiliations:** 1 Decision Modelling Center, Epidemiology & Data Science, location VUmc, Amsterdam University Medical Centres, Amsterdam, the Netherlands; 2 Department of Epidemiology, Julius Center for Health Sciences and Primary Care, University Medical Center Utrecht, Utrecht, the Netherlands; 3 Department of Medical Oncology, University Medical Center Utrecht, University Utrecht, Utrecht, the Netherlands

**Keywords:** metastatic colorectal cancer, capecitabine, S-1, Teysuno, fluoropyrimidines, oxaliplatin, i.v. 5-FU

## Abstract

**Background:**

The fluoropyrimidines 5-fluorouracil (5FU) and capecitabine are the backbone of systemic therapy for metastatic colorectal cancer (mCRC). Side effects include hand-foot syndrome (HFS) and cardiovascular toxicity (CVT), which may necessitate dose reductions or discontinuation of treatment. S-1 (Teysuno®) is an oral fluoropyrimidine that is licensed for use after fluoropyrimidine-induced HFS or CVT as it showed a lower incidence of those toxicities. It can be used as monotherapy or in combination with oxaliplatin or irinotecan. In this study, we assessed the cost-effectiveness of switching from 5FU or capecitabine-based treatment to S-1-based treatment after a patient with mCRC experiences HFS or CVT.

**Patients and methods:**

We developed a cohort-level decision analytic model to compare the costs and quality-adjusted life years (QALYs) when hypothetical patients would follow several different treatment strategies. We considered three different scenarios in which patients experienced toxicity on their initial first-line treatment of either CAPOX (1), FOLFOX (2) or capecitabine monotherapy (3), respectively. The step of first-line to second-line is denoted with an arrow (→). We used medication costs and administration costs, a lifelong time axis and compared strategies using incremental cost-effectiveness ratios (ICERs) and net benefit.

**Results:**

Costs for treatment administration often exceeded costs for treatment medication. For scenario 1, the ICER of the strategy of switching to SOX → Irinotecan (which was identical to SOX → IRIS) was €60 303 compared with the next best strategy of discontinuation of CAPOX → Irinotecan. This is below the threshold of €80 000/QALY used by the Dutch government for high-impact health conditions. Results of scenario 2 were similar to scenario 1. For scenario 3, the ICER of S-1 → SOX was €92 551 compared with reduced dosage capecitabine monotherapy → CAPOX.

**Conclusions:**

S-1 based treatment strategies can be cost-effective when a treatment switch is required due to HFS or CVT during fluoropyrimidine-based treatment.

## Highlights

• S-1 is an oral fluoropyrimidine approved for use in mCRC patients who cannot continue treatment due to HFS or CVT.
• S-1 showed lower incidences of HFS and CVT and allowed first-line treatment to immediately continue.
• The cost-effectiveness of S-1 is not yet researched.
• We developed a model to compare the costs and QALY for several different treatment strategies.
• We found that S-1 based treatment strategies can be cost-effective when a switch is required due to HFS or CVT.

## Introduction

Intravenous 5-fluorouracil (5FU) and oral capecitabine are fluoropyrimidines that are the backbone of first- and often second-line chemotherapy for patients with metastatic colorectal cancer (mCRC). Hand-foot syndrome (HFS) is a frequently occurring toxicity, occurring in up to 18% of patients treated with 5FU and 77% with capecitabine.^1^^,^^2^ Dose reductions are applied in patients who experience severe complaints of HFS. Despite dose reduction, HFS recurs in approximately one-third of patients, who require further dose reduction or discontinuation of treatment.^3^ Cardiovascular toxicity (CVT) is a life-threatening toxicity that occurs in 4% to 6% of patients receiving capecitabine or 5FU, after which treatment is usually permanently discontinued.^4^ 5FU and capecitabine may be administered as monotherapy or in combination with oxaliplatin or irinotecan, with or without targeted agents. The most frequently used chemotherapy combinations are capecitabine with oxaliplatin (CAPOX), 5FU with oxaliplatin (FOLFOX) and 5FU with irinotecan (FOLFIRI). Discontinuation of the fluoropyrimidine limits the treatment options for patients with mCRC, which may compromise their prognosis.^5^

S-1 (Tegafur/gimeracil/oteracil, Teysuno®) is an alternative oral fluoropyrimidine which was developed in Asian countries in the early 1990s for use in solid tumors.^6^ Later, the drug showed comparable efficacy but a favourable safety profile compared with capecitabine and 5FU, with a decreased occurrence of HFS and CVT being rare.^7^^,^^8^ This has since been confirmed in several studies based in Western countries, leading to approval of S-1 from the European Committee and European Medicines Agency as monotherapy or in combination with oxaliplatin (SOX) or irinotecan (IRIS) in mCRC patients who cannot continue 5FU/capecitabine-based treatment due to HFS or CVT, occurring either in the adjuvant or metastatic setting.^9^ The 2023 European Society for Medical Oncology guidelines for mCRC recommend S-1 as an alternative to dose reduction, dose delay or discontinuation of capecitabine and/or 5FU after HFS or CVT, and guidelines for its use in clinical practice have been formulated by an international expert panel.^5^^,^^10^ Retrospective studies have shown that a switch to S-1 in patients experiencing HFS of CVT is safe and feasible and allows treatment continuation at preferred dosages.^11^, ^12^, ^13^ The drug S-1 itself is more expensive than 5FU and capecitabine. However, S-1 is an oral drug like capecitabine, and does not require hospital-based administration of treatment and ambulatory infusion devices like 5FU requires, which may decrease the total treatment costs.

Our aim in this study was to assess the cost-effectiveness of switching to S-1 mono- or combination therapy after HFS or CVT have occurred during capecitabine or 5FU-based treatment of mCRC. To this end, we conducted a decision analytic modelling study.

## Methods

### Study overview

Our goal is to develop a cohort-level model (Markov model) to assess the costs and QALYs that mCRC patients accumulate over their lifetime, to help identify cost-effective treatment strategies.^14^ We will go through the steps of development one by one. For a more detailed explanation, please see the Supplementary Material I, available at https://doi.org/10.1016/j.esmoop.2026.106304, which includes an ‘example patient’ followed in the Markov model and calculations of accumulated costs, QALYs and net benefit.

### Population and decision problem

Our aim is to model the repercussions in terms of costs and QALYs for the treatment decision after HFS or CVT occurred. For the Markov model, our population of interest is therefore defined as mCRC patients who experienced HFS or CVT during their first-line treatment with capecitabine or 5FU-based regimens.

### Model scenarios and treatment strategies

Based on international guidelines and expert opinion (coauthors CJAP and JJMK), we found that three treatment pathways were the most common for chemotherapy in mCRC, namely starting with CAPOX in the first line followed by FOLFIRI in the second line, starting with FOLFOX followed by FOLFIRI and starting with capecitabine monotherapy followed by CAPOX.^10^

We distinguish between the terms ‘scenario’ and ‘strategy’: scenarios are the setting, here the first-line treatment that led to HFS or CVT is the ‘start’. Strategies are the combinations of first- and second-line treatment choices from then onward, for instance reduced dosage CAPOX followed by irinotecan monotherapy. In this paper, we use an arrow (→) for the step from first-line to second-line treatment, so the previous example would be denoted as reduced dosage CAPOX → irinotecan.

Note that when a patient experiences CVT, reduced dosage of capecitabine or 5FU is not a valid option due to the risk of recurrent CVT. However, for simplicity, we assume that strategies are identical for HFS and CVT, and for the latter, we simply ignore the strategy that incorporates reduced dosage first-line treatment (see ‘Sensitivity Analyses’ later in the text). For the second line, as FOLFIRI is not used after CVT, this option is replaced by irinotecan monotherapy. Finally, since bevacizumab may be used in any regimen (both before and after toxicity), their effects and costs are considered to cancel each other out and are not considered here.

Below, we go through the scenarios and define for each of them the treatment strategies for mCRC patients after occurrence of HFS or CVT.

In the first scenario, the patient started with CAPOX and then experienced toxicity. In the absence of toxicity, FOLFIRI would be the preferred second-line treatment. Given HFS or CVT, the available treatment strategies are:

discontinuation(untilprogression)→irinotecanmonotherapy

reduceddosageCAPOX→irinotecanmonotherapy

S-1 provides us with the additional option to switch first-line treatment, replacing capecitabine but keeping oxaliplatin as combination therapy (SOX). In addition, IRIS can be used as an S-1 based alternative for second-line treatment that includes irinotecan. This adds the strategies:

SOX→irinotecanmonotherapy

SOX→IRIS

These are the four strategies that we consider for this scenario.

In the second scenario, the patient started with FOLFOX and then experienced toxicity. In the absence of toxicity, FOLFIRI would be the preferred second-line treatment. Given HFS or CVT, the available treatment strategies are identical to the four identified for the first scenario except for CAPOX being changed to FOLFOX.

For the third scenario, patients started with full-dosage capecitabine monotherapy (1250 mg/m^2^) and experienced toxicity. In the absence of toxicity, CAPOX would be the preferred second-line treatment. Given HFS or CVT, the available treatment strategies are:

discontinuation(untilprogression)→irinotecanmonotherapy

reduceddosagecapecitabine→CAPOX

S-1 now provides us with an alternative monotherapy to switch first-line treatment that can be followed by SOX. This adds the strategy:

S-1→SOX

These are the three treatment strategies that we considered for this scenario.

Reduced dosage was 1000 mg/m^2^ for capecitabine and 1920 mg/m^2^ for 5FU. Standard dosage for S-1 monotherapy was 30 mg/m^2^ and for S-1 combination therapy was 25 mg/m^2^. The rest of the dosages were standard and are reported in detail in Supplementary Material II, available at https://doi.org/10.1016/j.esmoop.2026.106304.

### Overall design of the Markov model

The Markov model is a cohort-level model that follows a hypothetical cohort of 1000 patients through their treatment trajectory.^14^ Please see the Supplementary Material I, available at https://doi.org/10.1016/j.esmoop.2026.106304 for a more detailed explanation.

We considered first- and second-line treatment to be relevant for our research question, but not any treatment thereafter. We used five states: first-line treatment, progression, treatment after progression, no treatment after progression, and death. The time unit per Markov cycle was set to 1 week and the time scale to a lifetime approach, from the switch of the first-line treatment to death, with progression as a possible intercurrent event. The progression state lasted 1 week after which patients were divided into those who started second-line treatment (estimated at 80% of progressed patients) and those who did not (20% of progressed patients). Note that patients could still die during this week. We assumed that all second-line treatments had similar efficacy. Patients could die either before or after progression. Death is an absorbing state, meaning that a person remains in that state (i.e. reversal is not possible).

**Figure 1:**
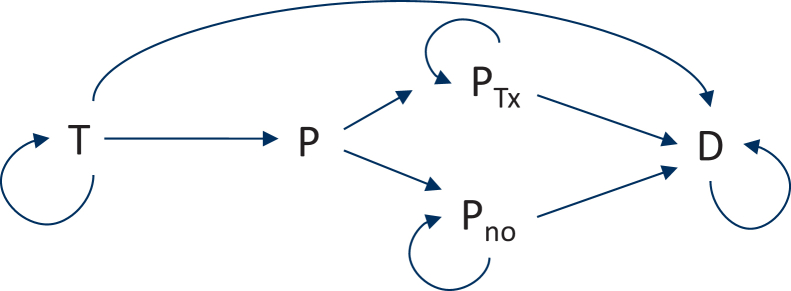
Overall structure of the Markov model. Arrows represent possible transitions, with an arrow pointing to its own state indicating that the patient, for that week, stays in that state.

The overall structure of the Markov model is shown in Figure 1. All strategies considered in the current study follow this general structure. Arrows represent possible transitions, with an arrow pointing to its own state indicating that the patient, for that week, stays in that state.

### Model parameters

Survival (time-to-death or overall survival, OS) and time-to-progression (TTP) parameters were based on data from the SALTO trial.^15^ We fitted exponential and Weibull models separately for OS and TTP and found that the Weibull model had a better fit in terms of a lower Akaike's Information Criterion than the exponential model. We thus used Weibull distributions for OS and TTP. We used non-parametric bootstrapping to estimate the correlation between the scale and shape parameters of the Weibull distribution. Note that these parameters describe the OS and TTP transition probabilities for the ‘base’ strategies that involve capecitabine or 5FU. Discontinuation changes the OS and TTP, and S-1-based strategies change the OS. These adjustments were conducted by applying relative risks to the transition probabilities.

Relative risks as treatment effect parameters were taken from the review and meta-analysis of Derksen et al. 2022.^8^ For OS, the estimated relative risk of S-1 versus capecitabine or intravenous-based (i.v.)-5FU was 0.93 [99% confidence interval (CI) 0.81-1.07]. For TTP, the relative risk was assumed to be 1.0 (i.e. no difference). The relative risk of both OS and TTP for treatment discontinuation (i.e. no first-line treatment) versus capecitabine or 5FU was set at 1.50 based on expert opinion (coauthors CJAP and JJMK), as no accurate data on untreated patients were available that did not suffer from severe confounding by indication (patients that waive first-line treatment tend to be those with the worst prognosis).^15^^,^^16^

The utility (quality-of-life; QoL) parameter for the starting health state (*T*) was based on the cancer-related European Organisation for Research and Treatment of Cancer Core Quality of Life questionnaire questionnaires filled out at baseline by participants from the CAIRO and CAIRO2 trials who received capecitabine monotherapy or CAPOX as first-line treatment, with standard errors of the means determined by non-parametric bootstrap.^17^^,^^18^ The average baseline QoL was 0.83 in both trials. We used the highest standard error of the two trials (0.01) for our uncertainty distribution.

We considered four adverse events as most important for these treatments in mCRC, two of which S-1 tends to decrease the probability that they will occur compared with capecitabine/5Fu and two of which S-1 tends to increase the probability that they will occur: recurrent HFS (grade ≥2), recurrent CVT (grade ≥1), diarrhoea and anorexia. For simplicity, as the timing was not crucial in our modelling approach, we assumed that adverse events occurred immediately at first-line treatment and lasted for 2 weeks. Disutilities (i.e. subtractions from utilities due to adverse events) were taken from Jeong et al., 2016, set at 0.15.^19^ The disutility for cardiotoxicity was not reported and was also set at 0.15. The disutility for the state of progressed disease and second-line treatment was based on Jeong et al. and expert opinion (coauthors RvE and VMHC), set at 0.15, with the disutility for the state progression without treatment being set at 0.10.^19^ Probabilities of the four adverse events for capecitabine/5FU or S-1 were fixed to values taken from a Dutch prospective cohort (Punt et al., 2022), the CardioSwitch study (Österlund et al., 2022) and data from the SALTO trial (Kwakman et al., 2017).^11^^,^^12^^,^^15^

### Costs

Direct medical costs involved the costs of chemotherapy medication, costs of administering medication (hospital admission and personnel, only applicable to i.v. medications) and costs of adverse events (HFS, CVT, diarrhoea and anorexia).

Medication costs were taken from online Dutch government pricing data (Zorginstituut Nederland, www.medicijnkosten.nl).^20^ Costs of administering medication were based on the Dutch government guideline for cost-effectiveness analyses.^21^ Note that costs for ambulatory 5FU administration were not available and difficult to estimate due to the many factors involved, for which Dutch standard tariffs are inaccurate. For transparency and robustness, we opted to use the reported estimates for hospital-based administration from the Dutch government guidelines instead. Costs of adverse events were estimated by expert opinion (CJAP and JJMK). All costs were indexed for 2024.

All costs, where relevant, were calculated per cycle for the average person with a body surface area of 1.95 (i.e. 177 cm and 78 kg), considered realistic for the Dutch average.^22^ Note that a treatment cycle with capecitabine or S-1 lasts 3 weeks and thus these were divided by 3, whereas a treatment cycle with 5FU lasts 2 weeks and thus these were divided by 2. All costs are reported in Table 1. Costs are reported in more detail in Supplementary Material II, available at https://doi.org/10.1016/j.esmoop.2026.106304, including additional details, dosages per cycle for all treatment regimens, averaging costs over all different drug dosages, etc.

**Table 1:** All model parameters and uncertainty distributions. Costs are in EUR

Parameter	Point estimate	Distribution	Comments or underlying assumptions	Source
Costs for S-1 (as monotherapy)	398 per cycle	$Gamma\left(\sqrt{398},\frac{1}{\sqrt{398}}\right)$	Costs for S-1 medication, 30 mg/m^2^	Dutch pharmacy costs (medicijnkosten.nl)
Costs for S-1 (in combination therapy)	331 per cycle	$Gamma\left(\sqrt{331},\frac{1}{\sqrt{331}}\right)$	Costs for S-1 medication, 25 mg/^2^	Dutch pharmacy costs (medicijnkosten.nl)
Costs for reduced capecitabine (both monotherapy and combined)	180 per cycle	$Gamma\left(\sqrt{180},\frac{1}{\sqrt{180}}\right)$	Costs for reduced dose capecitabine medication, 1000 mg/m^2^	Dutch pharmacy costs (medicijnkosten.nl)
Costs for oxaliplatin	556 per cycle	$Gamma\left(\sqrt{556},\frac{1}{\sqrt{556}}\right)$	Costs for oxaliplatin medication, 130 mg/m^2^	Dutch pharmacy costs (medicijnkosten.nl)
Costs for reduced dosage 5FU and leucovorin	208 per cycle	$Gamma\left(\sqrt{208},\frac{1}{\sqrt{208}}\right)$	Costs for 5FU and leucovorin medication, 400 mg/m^2^ initially then 2400 mg/m^2^	Dutch pharmacy costs (medicijnkosten.nl)
Costs for irinotecan (as monotherapy)	677 per cycle	$Gamma\left(\sqrt{677},\frac{1}{\sqrt{677}}\right)$	Costs for irinotecan medication, 350 mg/m^2^	Dutch pharmacy costs (medicijnkosten.nl)
Costs for irinotecan (in combination therapy)	348 per cycle	$Gamma\left(\sqrt{348},\frac{1}{\sqrt{348}}\right)$	Costs for irinotecan medication, 180 mg/m^2^	Dutch pharmacy costs (medicijnkosten.nl)
Costs for ‘short’ medication i.v. administration (2 hours)	335 per cycle	$Gamma\left(\sqrt{335},\frac{1}{\sqrt{335}}\right)$	Costs for ‘short’ administration of chemotherapy (oxaliplatin, irinotecan)	Dutch government (Kostenhandleiding 2024)
Costs for ‘long’ medication i.v. administration (48+ hours)	1288 per cycle	$Gamma\left(\sqrt{1288},\frac{1}{\sqrt{1288}}\right)$	Costs for ‘long’ administration of chemotherapy (5FU)	Dutch government (Kostenhandleiding 2024)
Total costs for CAPOX	320 per week	∑ofcomponents	Costs for reduced dosage capecitabine and oxaliplatin plus administration	Dutch pharmacy costs (medicijnkosten.nl) Dutch government (Kostenhandleiding 2024)
Total costs for FOLFOX	1026 per week	∑ofcomponents	Costs for reduced dosage 5FU + LV, oxaliplatin, and 48+ hours administration	Dutch pharmacy costs (medicijnkosten.nl) Dutch government (Kostenhandleiding 2024)
Total costs for SOX	407 per week	∑ofcomponents	Costs for S-1 and oxaliplatin medication plus administration	Dutch pharmacy costs (medicijnkosten.nl) Dutch government (Kostenhandleiding 2024)
Total costs for IRIS	338 per week	∑ofcomponents	Costs for S-1 and irinotecan medication plus administration	Dutch pharmacy costs (medicijnkosten.nl) Dutch government (Kostenhandleiding 2024)
Total costs for irinotecan (as monotherapy)	337 per week	∑ofcomponents	Costs for full-dosage irinotecan medication plus administration	Dutch pharmacy costs (medicijnkosten.nl) Dutch government (Kostenhandleiding 2024)
Costs for recurrence of HFS	50		Costs for creams, inconvenience	Expert opinion
Costs for recurrent CT	200		Costs for treatment disruption, hospitalisation	Expert opinion
Relative risk of death for S-1 versus capecitabine or i.v.-5FU	0.93	Lognormal (−0.0726, 0.058)	Capecitabine and i.v.-5FU are interchangeable in their effects	Derksen et al. (2022)^8^
Relative risk of death for no treatment versus S-1, capecitabine or i.v.-5FU	1.50		No treatment decreases the time-to-death and time-to-progression Sensitivity analysis	Expert opinion
Proportion of progressed patients who receive second-line treatment	0.80		In combination with time-to-progression as estimated in the SALTO trial	Kwakman et al. (2017)^4^^,^^15^ Kwakman et al. (2019)^23^
Proportion of patients that suffer from recurrent HFS after switch	Capecitabine/IV5FU: 0.55 S-1: 0.05		Retrospective cohort on switchers and estimated in SALTO trial	Punt et al. (2022)^11^ Kwakman et al. (2017)^4^^,^^15^ Kwakman et al. (2019)^23^
Proportion of patients that suffer from recurrent CT after switch	Capecitabine/IV5FU: 0.40 S-1: 0.04		CardioSwitch cohort and estimated in SALTO trial	Österlund et al. (2022)^12^ Kwakman et al. (2017)^4^^,^^15^ Kwakman et al. (2019)^23^
Quality of life (first-line)	0.83	N (0.83, 0.01)	CAIRO and CAIRO2 studies	Koopman et al. (2007)^17^ Tol et al. (2009)^18^
Quality of life (second-line treatment)	First-line QoL - 0.15		Disease has progressed	Jeong and Cairns (2016)^19^
Quality of life (no second-line treatment)	First-line QoL - 0.10		Quality of life is slightly higher as there is no burden of treatment	
Quality of life (death)	0		Worst possible quality of life	
Disutility for adverse events	0.15		Disutility is applied for 3 consecutive weeks	Jeong and Cairns (2016)^19^
Time-to-death (for capecitabine, CAPOX, or i.v.-5FU)	Weibull (1.473,79.4)	Shape: N (1.473,0.096) Scale: N (79.4,4.874) Correlation shape-scale: 0.555	Estimated in SALTO trial	Kwakman et al. (2017)^4^^,^^15^ Kwakman et al. (2019)^23^
Time-to-progression (for capecitabine, CAPOX, i.v.-5FU or S-1 based strategies)	Weibull (1.560,53.9)	Shape: N (1.560,0.107) Scale: N (53.9,3.243) Correlation shape-scale: −0.255	Estimated in SALTO trial	Kwakman et al. (2017)^4^^,^^15^ Kwakman et al. (2019)^23^
Discount rates:	Fixed: 3% (costs) Fixed: 1.5% (utility)		Costs or health effects paid or gained immediately are more important than those paid or gained later	Dutch government (Kostenhandleiding 2024)

CT/CVT, cardiovascular toxicity; HFS, hand-foot syndrome; i.v.-5FU, intravenous fluorouracil; N, normal.

### Uncertainty quantification

We incorporated the uncertainty in the parameters in our Markov model by running the model 1000 times.^14^ For every run, we drew the value of a parameter from its uncertainty distribution [i.e. a probabilistic sensitivity analysis (PSA)]. Whenever possible, uncertainty distributions were taken or estimated given the source (paper, study or data) where the parameter was from. Uncertainty distributions of cost parameters were gamma distributions with the square root of the cost as the shape and the inverse of the square root of the mean as the rate.

Model parameters and the uncertainty distributions are reported in Table 1.

### Comparing strategies

Costs and effects were compared using the incremental cost-effectiveness ratio (ICER), dividing the difference in costs by the difference in effects (QALYs). We then compared strategies that became increasingly more expensive but also more effective.

Because there were multiple treatment strategies to compare, the ICER is somewhat difficult to interpret, as it involves multiple comparisons between pairs of increasingly more expensive options. To facilitate interpretation and allow for simultaneous comparison of all strategies, we used the net benefit framework.^14^ Using this framework, we first set ‘1 QALY’ at a monetary value ranging from €0 to €100 000 (the willingness-to-pay threshold, WTP). Next, for each strategy, we multiplied the QALYs gained with this value and subtracted the costs of said strategy. The result is the net benefit in terms of monetary gains for each strategy, given the WTP (i.e. the value 1 QALY is deemed to be worth). We then find which strategy yields the highest net benefit. We ran this algorithm over all 1000 PSA runs for the range of the WTP threshold from €0 to €100 000 per QALY. From the results, we can derive a net benefit curve that shows the proportion [i.e. the probability of each strategy yielding the highest net benefit (y-axis), given the WTP for 1 QALY (x-axis)].

### Perspective and discounting

We opted for the health care perspective, considering direct medical costs.

We included discounting in our lifetime timescale, using annual discount rates of 1.5% for utilities and 3% for costs in accordance with the Dutch government guideline for cost-effectiveness analyses.^21^

### Sensitivity analyses

We assessed the influence of some of our main assumptions on the results.

As we have fixed the relative risk for time-to-death and TTP to 1.5 for strategies in which first-line treatment is discontinued (versus capecitabine or 5FU), we assessed what the results would be if we set the relative risk lower (to 1.2) or higher (to 1.8).

Next, we described our target population as a combination of patients who experienced CVT and patients who experienced HFS. Although for this cost-effectiveness analysis these are very similar, there are two differences: (i) not all strategies are relevant options for both, which means that the reduced dosage first-line treatment can be ignored from the CVT perspective; (ii) HFS tends to occur later, after approximately three full cycles (9 weeks). To model this scenario in a sensitivity analysis, we estimated our Weibull parameters for OS in the SALTO trial data on patients who survived 9 weeks, then reran the model using these parameters.

### Software

The Markov model was programmed in R 4.2.0 and RStudio, using the *heemod* package.^24^

## Results

### Pricing

All costs are reported in Table 1. Note that some costs are denoted per treatment cycle and some are denoted per week, as oral drugs follow a 3-week cycle and i.v.-based drugs follow a 2-week cycle. Costs are explained in more detail (including dosages) in the Supplementary Material II, available at https://doi.org/10.1016/j.esmoop.2026.106304.

Drug prices for standard dosages per treatment cycle (2 or 3 weeks) were €180 for reduced dose capecitabine monotherapy or combination therapy, €208 for 5FU plus leucovorin, €398 for S-1 monotherapy, €331 for S-1 combination therapy, €556 for oxaliplatin, €677 for irinotecan monotherapy and €348 for irinotecan combination therapy. Prices for administration of chemotherapy per cycle were set at €0 for oral medication (capecitabine and S-1) but costs for i.v. medication were set at €335 for those administered within a short period (oxaliplatin and irinotecan, ∼2 hours) and €1288 for those administered over long periods (5FU, ∼2 days).

This yielded total costs per time unit of 1 week of €60 for reduced dose capecitabine monotherapy, €357 for CAPOX, €1026 for reduced dose FOLFOX, €337 for irinotecan monotherapy, €133 for S-1 monotherapy, €407 for SOX, and €338 for IRIS.

### Survival parameters and an example timeline

Supplementary Figure S1A and B, available at https://doi.org/10.1016/j.esmoop.2026.106304 shows the survival curves for OS (A) and TTP (B) from the SALTO trial data. Based on the exponential model fit (red line) and Weibull model fit (green line) compared with the Kaplan–Meier estimate (black line), we showed that both visually and in terms of the lower Akaike's Information Criterion, the Weibull model showed a better fit.

The median OS was 14.8 months and the median TTP was 8.9 months, which corresponds to ∼64 and ∼38 weeks.

For the timeline and results of a hypothetical patient in our Markov model, please see the paragraph in Supplementary Material I, available at https://doi.org/10.1016/j.esmoop.2026.106304.

### Model results

The results averaged over the PSA runs for scenario 1 in which patients started with CAPOX are presented in Table 2. Average QALYs per patient were lowest for the strategy involving discontinuation (0.80) and increased for reduced dosage CAPOX (1.04) and SOX strategies (1.10). Given identical QALYs and a difference in total costs of €41, which is negligible given the uncertainty in our model, we considered SOX → irinotecan and SOX → IRIS to be equivalent strategies that showed similar results. The ICER for reduced dosage CAPOX → irinotecan compared with the discontinuation strategy was €62 288. The ICER for SOX → irinotecan (or the equivalent SOX → IRIS) compared with the discontinuation strategy was €60 303. Thus, due to the latter having a lower ICER, reduced dosage CAPOX → irinotecan was weakly dominated by SOX → irinotecan, known as extended domination.

**Table 2:** Results from the Markov model averaged over probabilistic sensitivity analysis runs

Strategy	Total costs per patient (€, EUR)	Costs first-line (€)	Costs second-line (€)	Costs AEs (€, EUR)	Average QALYs per patient	Median OS (months)	Median time-to-progression (months)	Average number of first-line treatment cycles	Average number of second-line treatment cycles	Percentage of patients with progression	ICER (€ per 1 additional QALY)
Stop → irinotecan	6910	0	6910	0	0.80	11.1	10.0	0	6.7	63	Reference
Reduced CAPOX → irinotecan	22 112	12 842	9163	107	1.04	14.5	12.8	12.1	8.9	64	62 288 (weakly dominated)
SOX → irinotecan	24 919	14 957	9952	10	1.10	15.3	12.5	12.2	9.6	65	60 303
SOX → IRIS	24 960	14 957	9992	10	1.10	15.3	12.5	12.2	9.9	65	Equivalent

In this scenario, patients started CAPOX as first-line treatment after metastatic colorectal cancer diagnosis.

The results for scenario 2 in which patients started with FOLFOX are presented in Table 3. Results were like those in scenario 1, although reduced dosage FOLFOX was much more expensive compared with SOX-based first-line options and was thus dominated, meaning that the strategies involving SOX were less expensive while also yielding slightly more QALYs. The ICER for SOX → irinotecan (or the equivalent SOX → IRIS) compared with discontinuation → irinotecan was €60 534.

**Table 3:** Results from the Markov model averaged over probabilistic sensitivity analysis runs

Strategy	Total costs per patient (€)	Costs first-line (€)	Costs second-line (€)	Costs AEs (€, EUR)	Average QALYs per patient	Median OS (months)	Median time-to-progression (months)	Average number of first-line treatment cycles	Average number of second-line treatment cycles	Percentage of patients with progression	ICER (€ per 1 additional QALY)
Stop → irinotecan	7044	0	7044	0	0.80	11.1	10.0	0	6.7	63	Reference
Reduced FOLFOX → irinotecan	46 569	37 299	9163	107	1.04	14.5	12.8	18.3	8.9	64	Dominated
SOX → irinotecan	25 169	15 197	9952	10	1.10	15.3	12.5	12.2	9.6	65	60 534
SOX → IRIS	25 179	15 207	9992	10	1.10	15.3	12.5	12.2	9.9	65	Equivalent

In this scenario, patients started FOLFOX as first-line treatment after metastatic colorectal cancer diagnosis.

The results for scenario 3 in which patients started with capecitabine monotherapy are presented in Table 4. Average QALYs were identical to the previous scenarios although costs were lower because the first-line treatments were oral monotherapies. The ICER for reduced capecitabine → CAPOX versus discontinuation → irinotecan was €21 076. The ICER for S-1 → SOX versus reduced capecitabine → CAPOX was €92 551.

**Table 4:** Results from the Markov model averaged over probabilistic sensitivity analysis runs

Strategy	Total costs per patient (€)	Costs first-line (€)	Costs second-line (€)	Costs AEs (€)	Average QALYs per patient	Median OS (months)	Median time-to-progression (months)	Average number of first-line treatment cycles	Average number of second-line treatment cycles	Percentage of patients with progression	ICER (€ per 1 additional QALY)
Stop → irinotecan	6992	0	6992	0	0.80	11.1	10.1	0	6.9	63	Reference
Reduced CAP → CAPOX	12 123	2209	9810	104	1.04	14.6	12.9	12.3	9.2	64	21 076
S-1 → SOX	17 012	4904	12 098	10	1.10	15.3	12.6	12.4	9.9	65	92 551

In this scenario, patients started capecitabine monotherapy as first-line treatment after metastatic colorectal cancer diagnosis.

ICER, incremental cost-effectiveness ratio; PSA, probabilistic sensitivity analysis.

### Curves of the probability of net benefit

**Figure 2:**
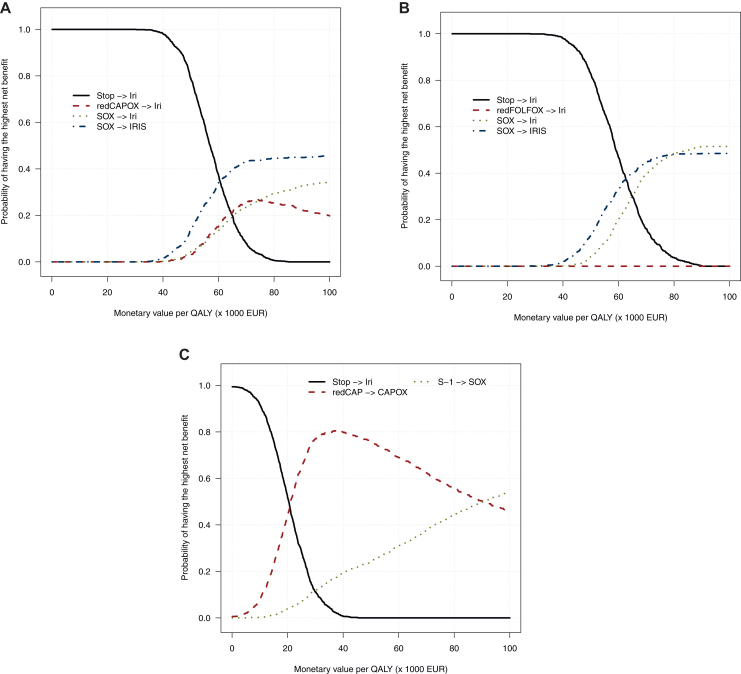
**(A-C) Net benefit curves for the three scenarios.** The y-axis represents the probability that a given strategy yields the highest net benefit, estimated as the proportion of probabilistic sensitivity analysis runs in which that strategy had the highest net benefit. The x-axis represents the value of 1 QALY, which we ranged from €0 to €100 000 (willingness-to pay, WTP). Iri, irinotecan; redCAP, reduced capecitabine monotherapy; redCAPOX, reduced CAPOX; redFOLFOX, reduced FOLFOX; Stop, discontinuation of first-line treatment.

The net benefit curves for scenario 1 in which patients experienced toxicity on CAPOX is shown in Figure 2A. We see that when we set the WTP for 1 QALY at €0 (i.e. nothing), the least expensive strategy is logically always the most likely to yield the highest net benefit. As we increase WTP (i.e. slide along the x-axis to the right), other strategies become more likely to yield the highest net benefit, with the turning point at a WTP of €60 000.

The net benefit curves for scenario 2 in which patients experienced toxicity on FOLFOX is shown in Figure 2B, which were identical to scenario 1 except for reduced FOLFOX never yielding the highest net benefit as it was the most expensive strategy by a large margin.

The net benefit curves for scenario 3 in which patients experienced toxicity on capecitabine monotherapy is shown in Figure 2C. Between €0 and €20 000, the strategy of discontinuation → irinotecan yielded the highest net benefit, between €20 000 and €90 000 this was reduced dosage capecitabine monotherapy → CAPOX and above €90 000 this was S-1 → SOX.

### Sensitivity analyses

Results for the sensitivity analysis in which we changed the relative risk of no treatment versus capecitabine/5FU from 1.5 to 1.2 is shown in Supplementary Table S1A-C and Figure S2A-C, available at https://doi.org/10.1016/j.esmoop.2026.106304. Results for the sensitivity analysis in which we changed the relative risk of no treatment versus capecitabine/5FU from 1.5 to 1.8 is shown in Supplementary Table S2A-C and Figure S3A-C. As expected, the strategy involving treatment discontinuation was better when we changed the relative risk to 1.2 and worse when we changed the relative risk to 1.8. The ICER for scenario 1 of SOX → irinotecan increased to €99 546 for the former and decreased to €48 190 for the latter. Scenario 2 was again similar to scenario 1. Scenario 3 did not change compared with the primary analysis.

The sensitivity analysis in which we used the OS parameters from the SALTO trial estimated after 9 weeks (i.e. three full cycles of capecitabine) is shown in Supplementary Table S3A-C and Figure S4A-C, available at https://doi.org/10.1016/j.esmoop.2026.106304. Results were similar to the primary analysis.

## Discussion

In this study, we assessed the cost-effectiveness of switching to S-1 mono- or combination therapy after HFS or CVT occurred using capecitabine or 5FU in the treatment of mCRC. We modelled the QALYs and total costs for three scenarios and various treatment strategies.

For the first scenario where patients experienced HFS or CVT on CAPOX, SOX → irinotecan and SOX → IRIS were identified as the cost-effective strategies with an ICER of €60 303 compared with discontinuation → irinotecan. For the second scenario where patients experienced toxicity on FOLFOX, the cost-effective strategy was SOX → irinotecan or SOX → IRIS, with an ICER of €60 534 compared with discontinuation → irinotecan. For the third scenario where patients experienced toxicity on capecitabine monotherapy, the strategy yielding the most QALYs was S-1 → SOX, with an ICER of €92 551 compared with reduced dosage capecitabine → CAPOX.

At the threshold of €80 000 per QALY used by the Dutch government for health conditions that are considered to be high impact (i.e. a high burden of disease), the S-1 based treatment strategies were cost-effective (or around the threshold), allowing treatment to continue immediately at full dosage.^25^ For all three scenarios there was a reasonable amount of uncertainty around the ICERs and the WTPs but it was clear that treatment discontinuation yielded the lowest QALYs. The threshold for WTP may differ based on the stakeholders’ perspective, country, etc. and may not be equivalent to the Dutch threshold of €80 000 per QALY we used here. However, our results provide the necessary information to test whether a strategy would be cost-effective for different WTPs. For instance, based on the British National Institute of Health and Care Excellence threshold of £30 000 per QALY (∼€34 000 per QALY), none of the S-1-based strategies would be considered cost-effective.^26^

To our knowledge, this is the first cost-effectiveness analysis that incorporates S-1-based options. A key finding was that chemotherapy administration in hospitals was the most important cost parameter, responsible for the largest difference in costs between treatment strategies. This holds especially for 5FU, which may require 48-hour hospitalization that costs 28 times as much as the medication itself (or 5 times as much when including leucovorin), making it also significantly more expensive than 2-hour administration of oxaliplatin or irinotecan. Although ambulant administration of 5FU may reduce costs compared with hospital-based administration, these costs remain substantially higher than the other options. Oral monotherapy options (i.e. capecitabine and S-1) avoid administration costs entirely.

Our findings might not be generalisable to all settings, especially those that use treatment strategies that we did not consider in our model and settings in which the medication costs and/or the treatment administration costs might differ. However, we hope our modelling approach, in which the explicit choice between switching or discontinuing treatments after toxicity is considered, provides the basis that one can use to reason if in their setting S-1 would likely to be cost-effective.

The Markov model used in this study combined the evidence from several contemporary sources to provide nuanced and detailed projections for various patient pathways over a lifetime horizon, although it is important to note that these still represent estimates that relied on imperfect data and modelling assumptions. The PSA allows for critical appraisal of our findings in the presence of these imperfect, sometimes uncertain data.

There remain some important uncertainties in this study, most notably the expected relative risks regarding OS and TTP for strategies that discontinued first-line treatment. We found in the sensitivity analyses that this parameter was important for results, as treatment discontinuation is the least expensive option, and as such it is the benchmark against which all strategies incorporating first-line treatment are compared. The higher the relative risk, the better first-line treatments will be, relative to discontinuing. Unfortunately, reports of survival and/or TTP after treatment discontinuation in literature are heavily biased, as the most common reason to stop or withhold from treatment is due to low life expectancy. We found in the SALTO trial that the median time-to-death of 15 months dropped to 4 months in patients who discontinued treatment. Using those data would clearly lead to a relative risk that is biased upward. We still consider our estimate of 1.50 to be on the conservative side, but we note that this principle is important for the interpretation of our results. Another important uncertainty was the comparative effectiveness of all treatment options for mCRC in first- and second-line treatment, which could not fully be implemented. Finally, as the first cost-effectiveness analysis study to incorporate S-1 in its strategies, we could not make a comparison to other cost-effectiveness analysis- or modelling studies.

In conclusion, our study showed that although S-1 is more expensive than capecitabine per week of treatment, it may reduce costs compared with 5FU-based options due to savings from reduced treatment administration time. Moreover, we have shown that S-1-based treatment strategies can be cost-effective when a treatment switch is required due to HFS or CVT during fluoropyrimidine-based treatment. Following the European Medicines Agency approval and incorporation in the 2023 European Society of Medical Oncology guidelines for mCRC, this positions S-1 as an attractive option for health care systems aiming to lower treatment costs while reducing toxicity and maintaining quality and efficacy.
