# Supplementary material for: Cost-effectiveness of switching to S-1 after fluoropyrimidine-induced hand-foot syndrome or cardiovascular toxicity in the treatment of metastatic colorectal cancer
Source: ESMO Open. 2026 Mar 17;11(4):106304. doi: 10.1016/j.esmoop.2026.106304 (PMC13015576; doi:10.1016/j.esmoop.2026.106304)
Supplement: Supplementary Figures [file mmc1.docx]

**Supplementary Figures**

**
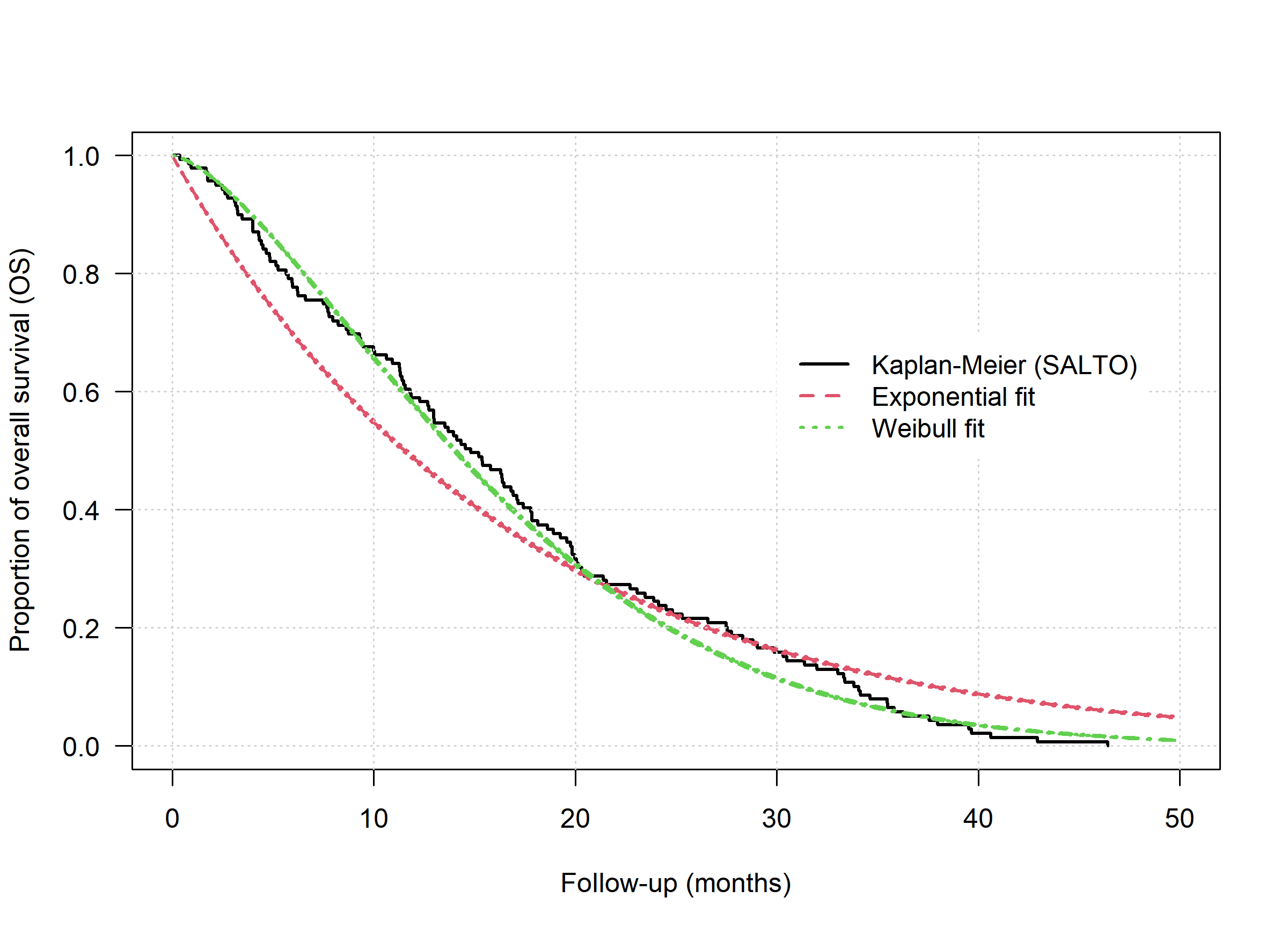
Supplementary Figure 1A and 1B. Survival curves for OS (A) and time-to-progression (B) from the SALTO trial.**

**A.**

**
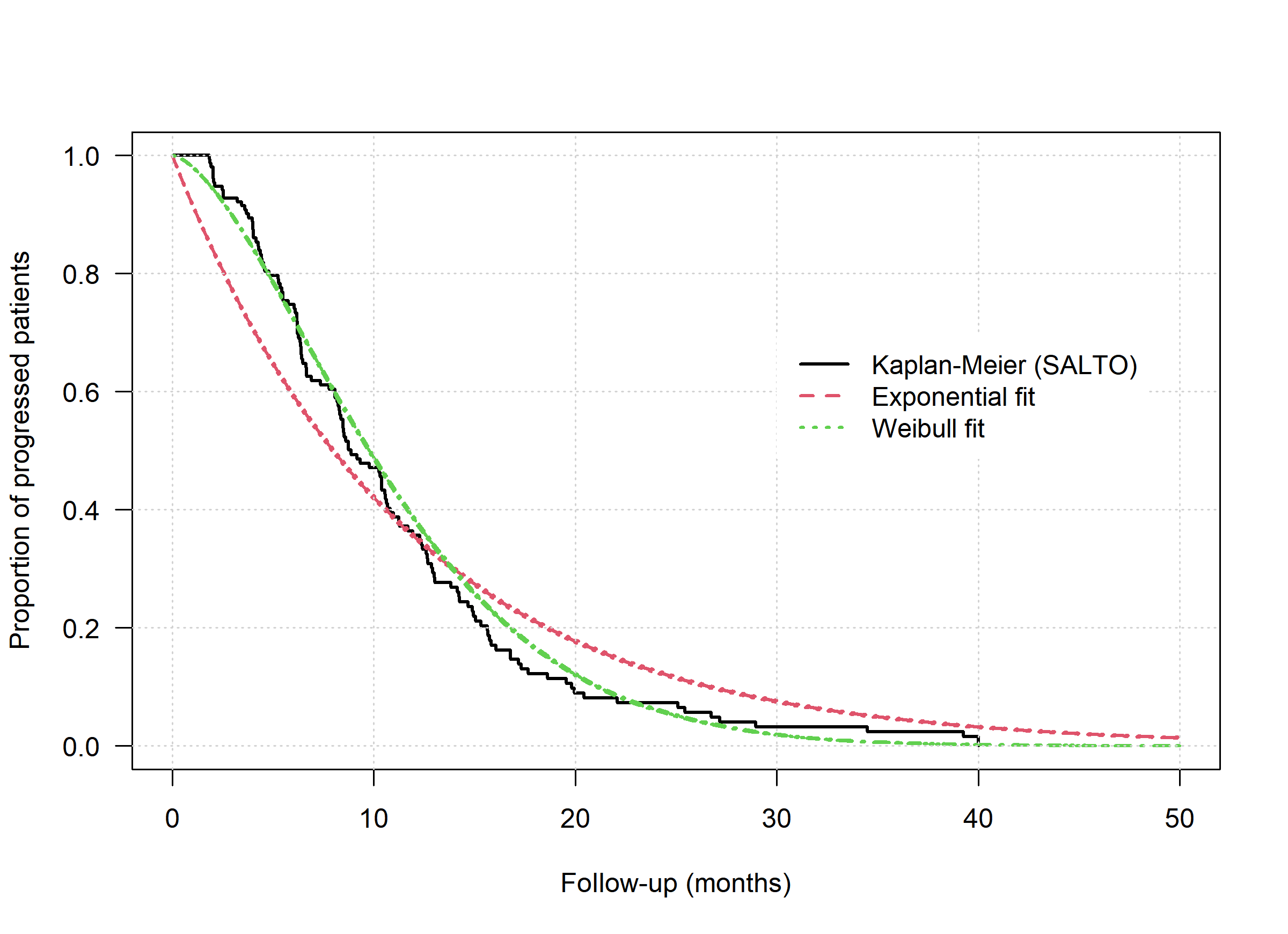
**

**B.**

**Supplementary Figures 2A-2C. Net benefit curves for the first sensitivity analysis, with a relative risk of 1.2 for no treatment vs. capecitabine/IV-5FU (instead of 1.5).**

1.
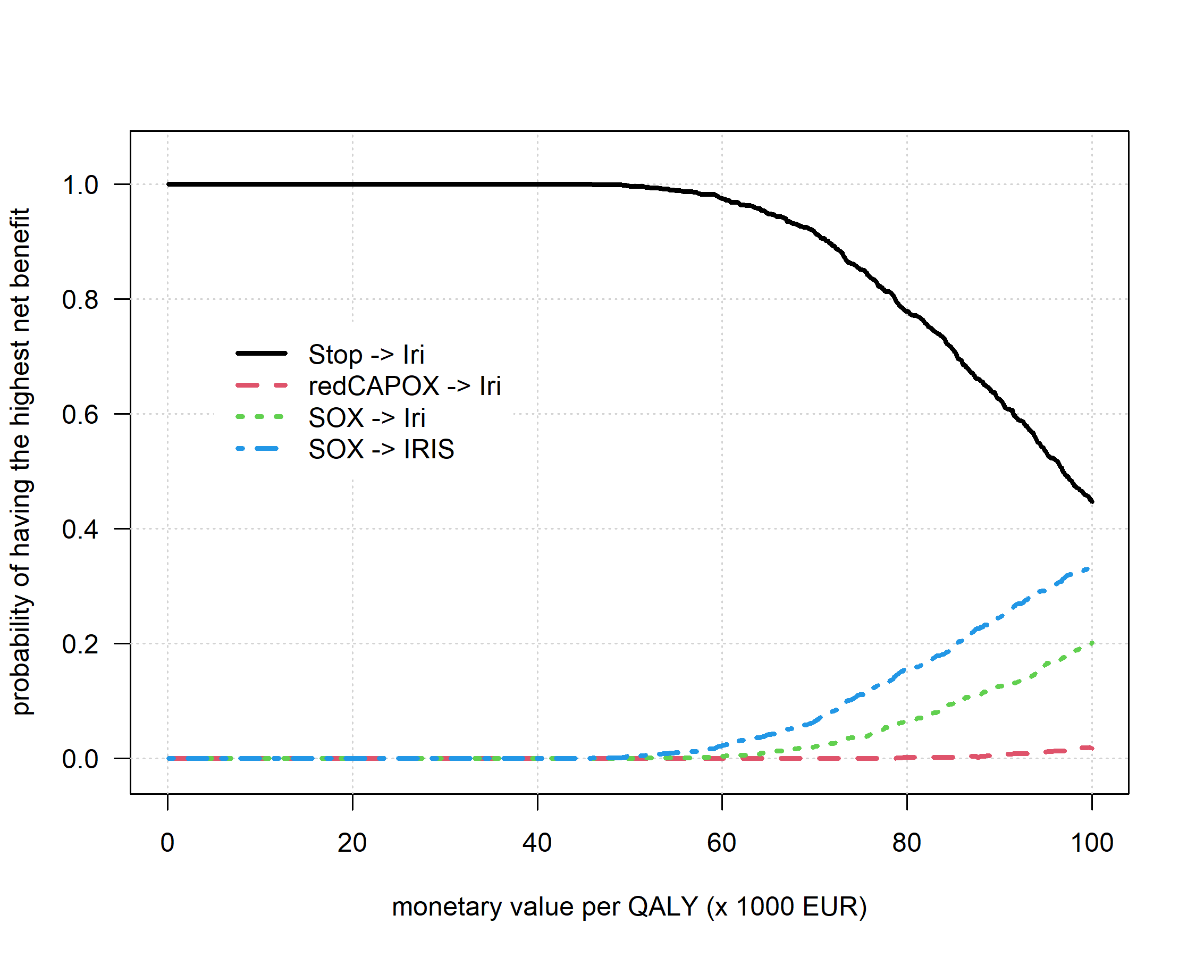
**Net benefit curve for the scenario where patients started with CAPOX.**


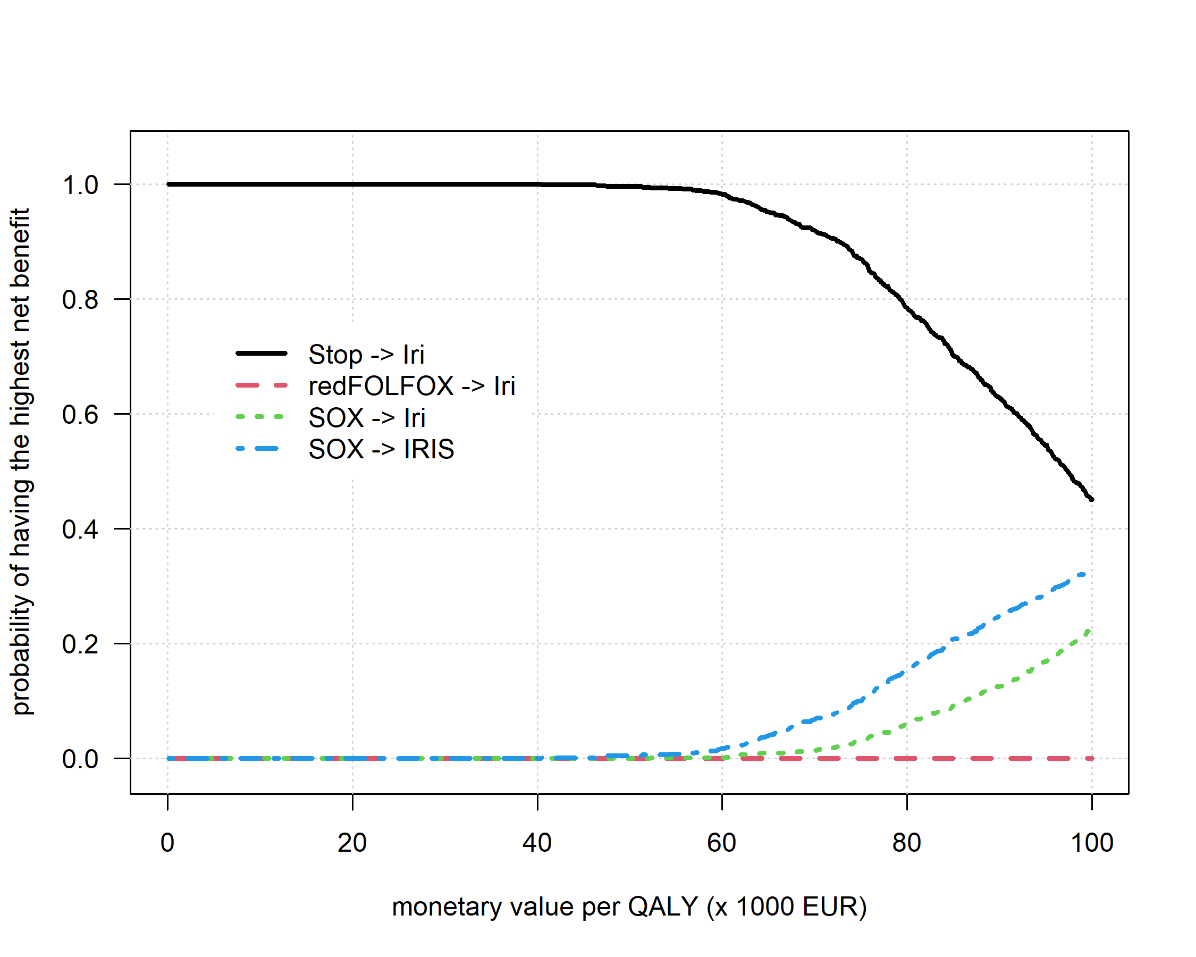
**B. Net benefit curve for the scenario where patients started with FOLFOX.**

**C. Net benefit curve for the scenario where patients started with capecitabine.**


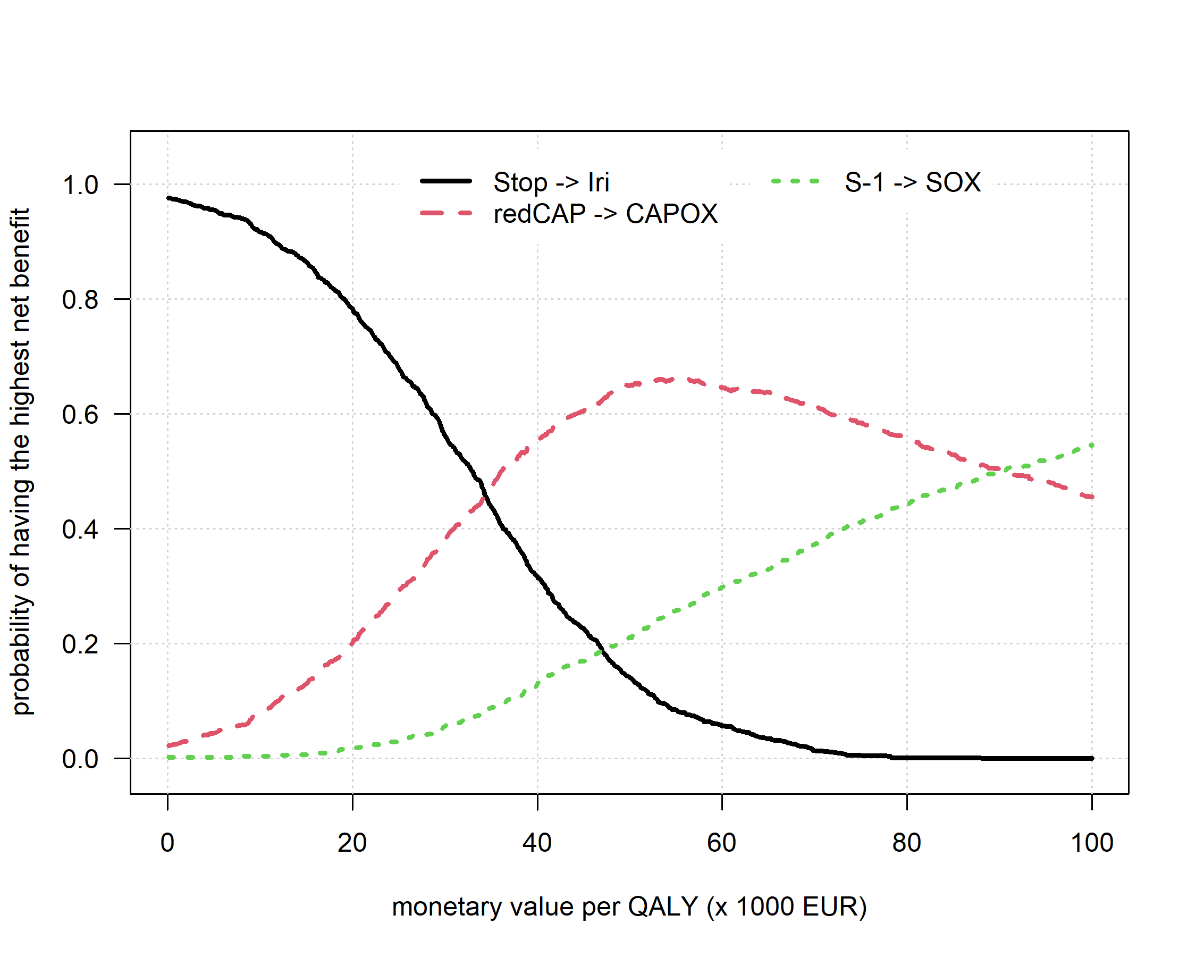


**Supplementary Figures 3A-3C. Net benefit curves for the first sensitivity analysis, with a relative risk of 1.8 for no treatment vs. capecitabine/IV-5FU (instead of 1.5).**


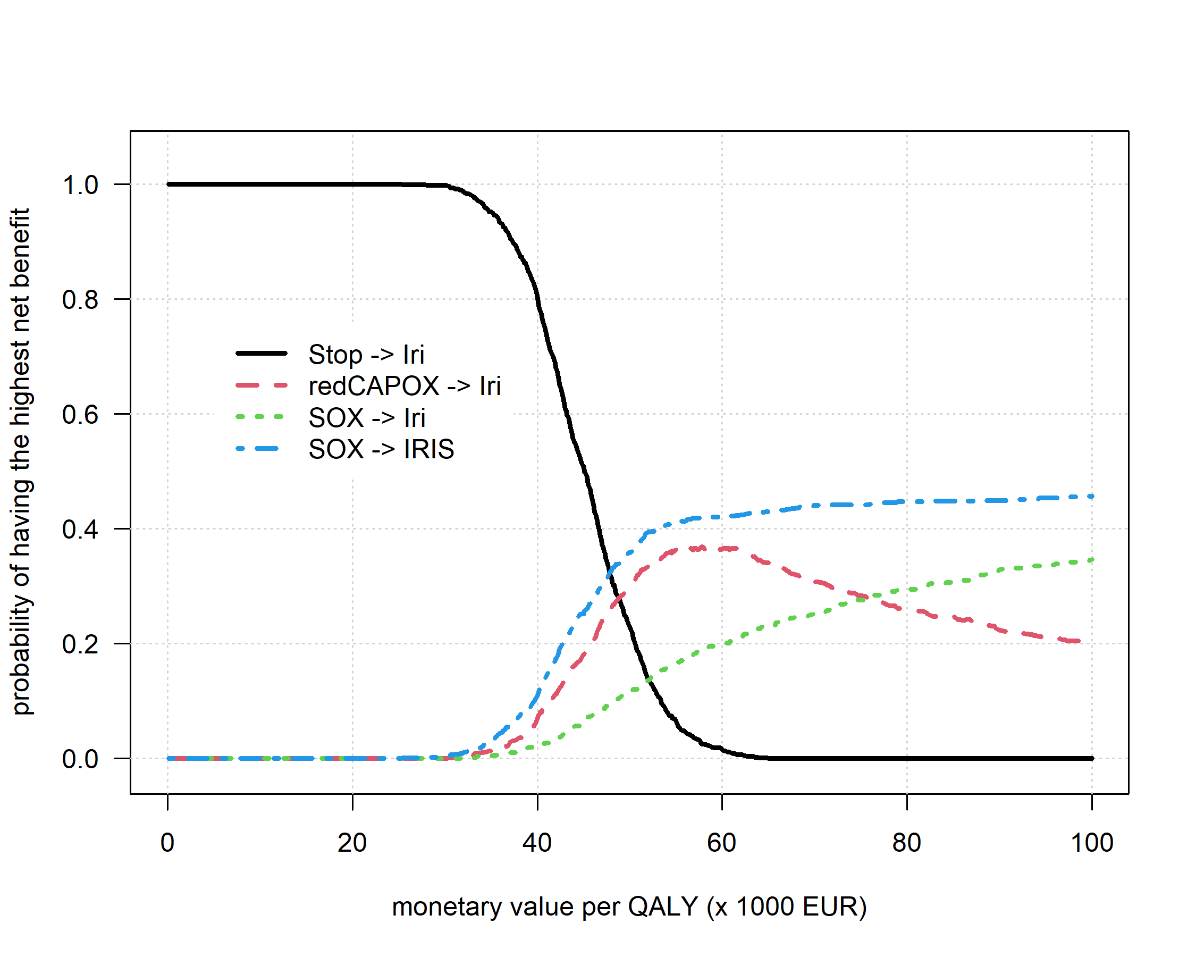
**A. Net benefit curve for the scenario where patients started with CAPOX.**


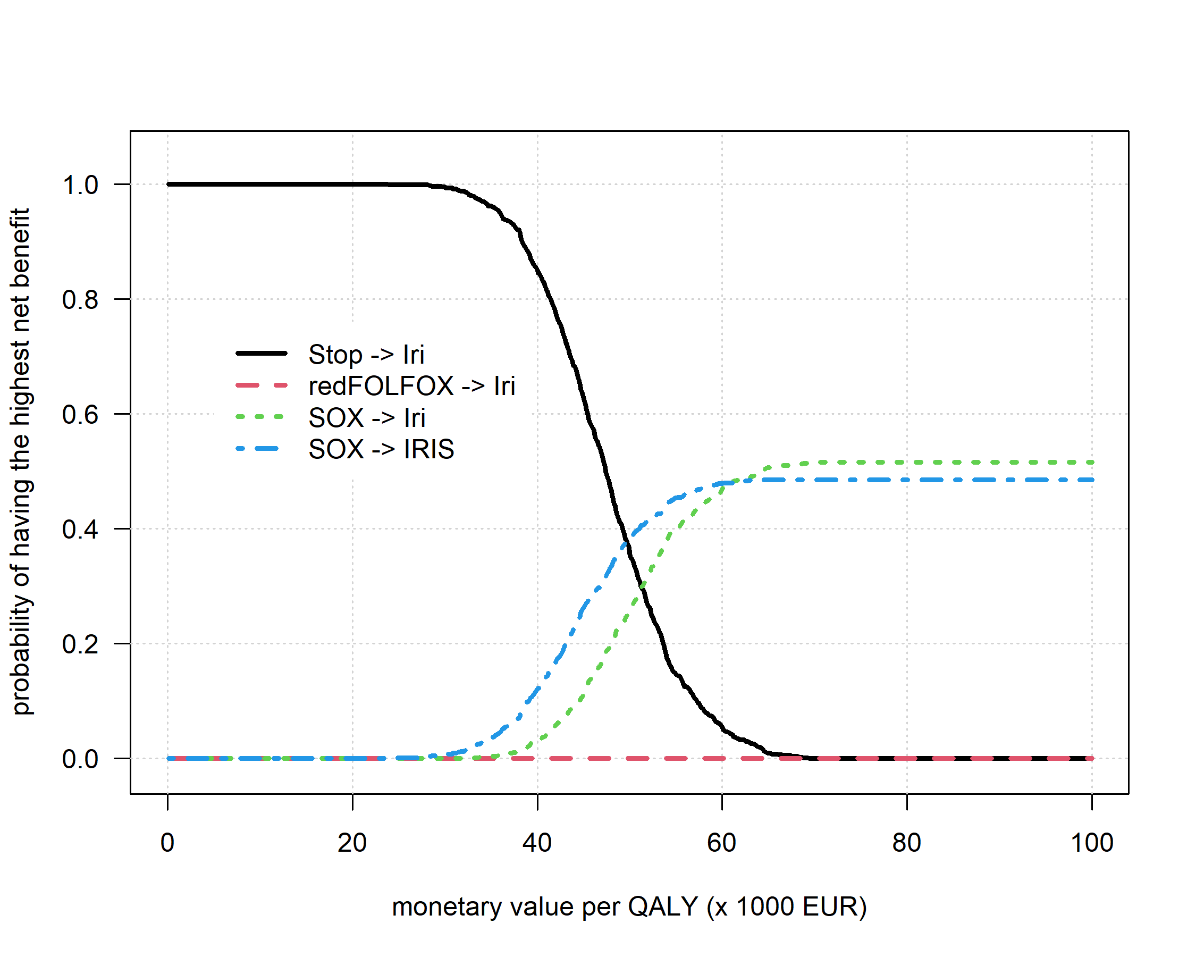
**B. Net benefit curve for the scenario where patients started with FOLFOX.**

**C. Net benefit curve for the scenario where patients started with capecitabine.**


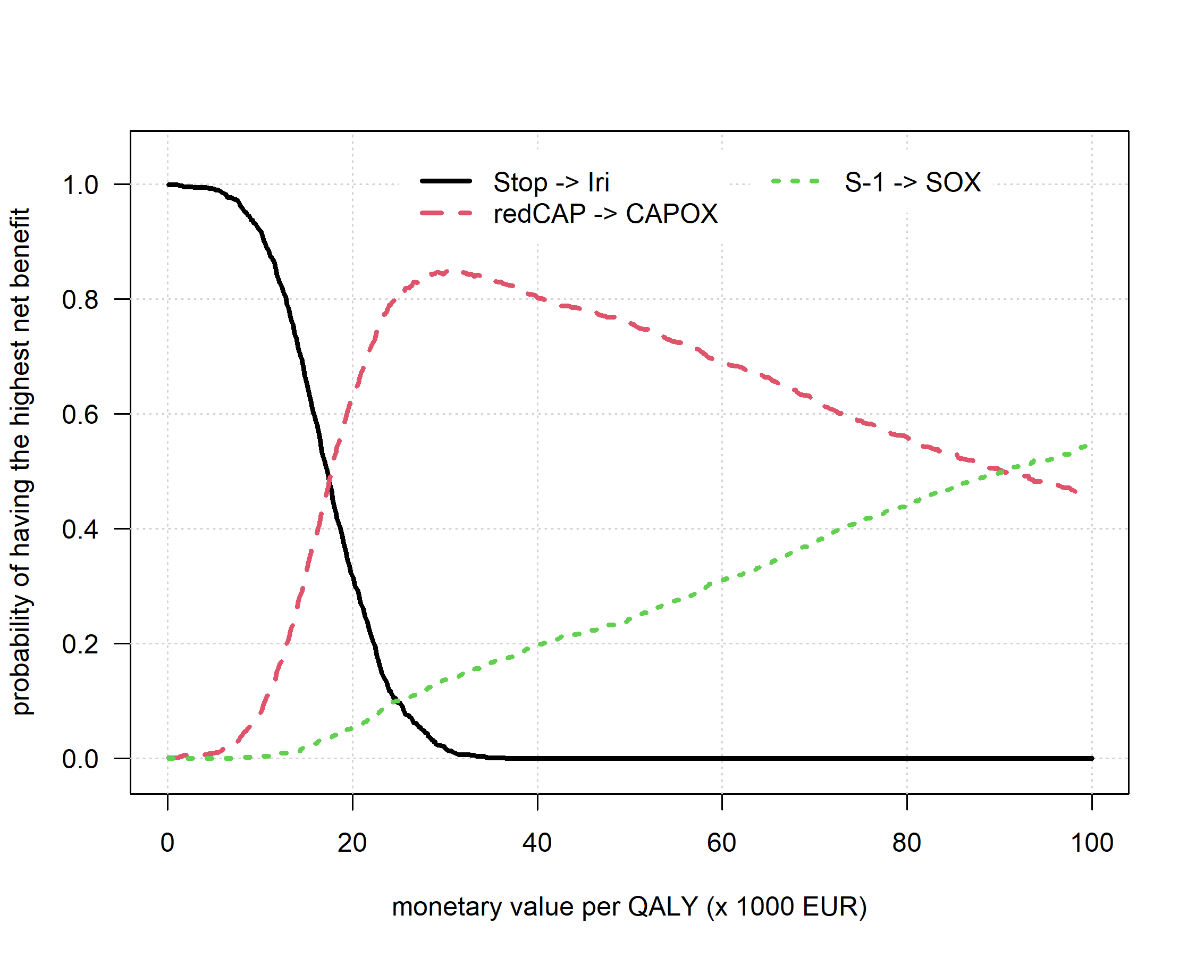


**Supplementary Figures 4A-4C. Net benefit curves for the second sensitivity analysis using survival data from the SALTO trial for patients after 9 weeks (instead of after randomisation).**

1.
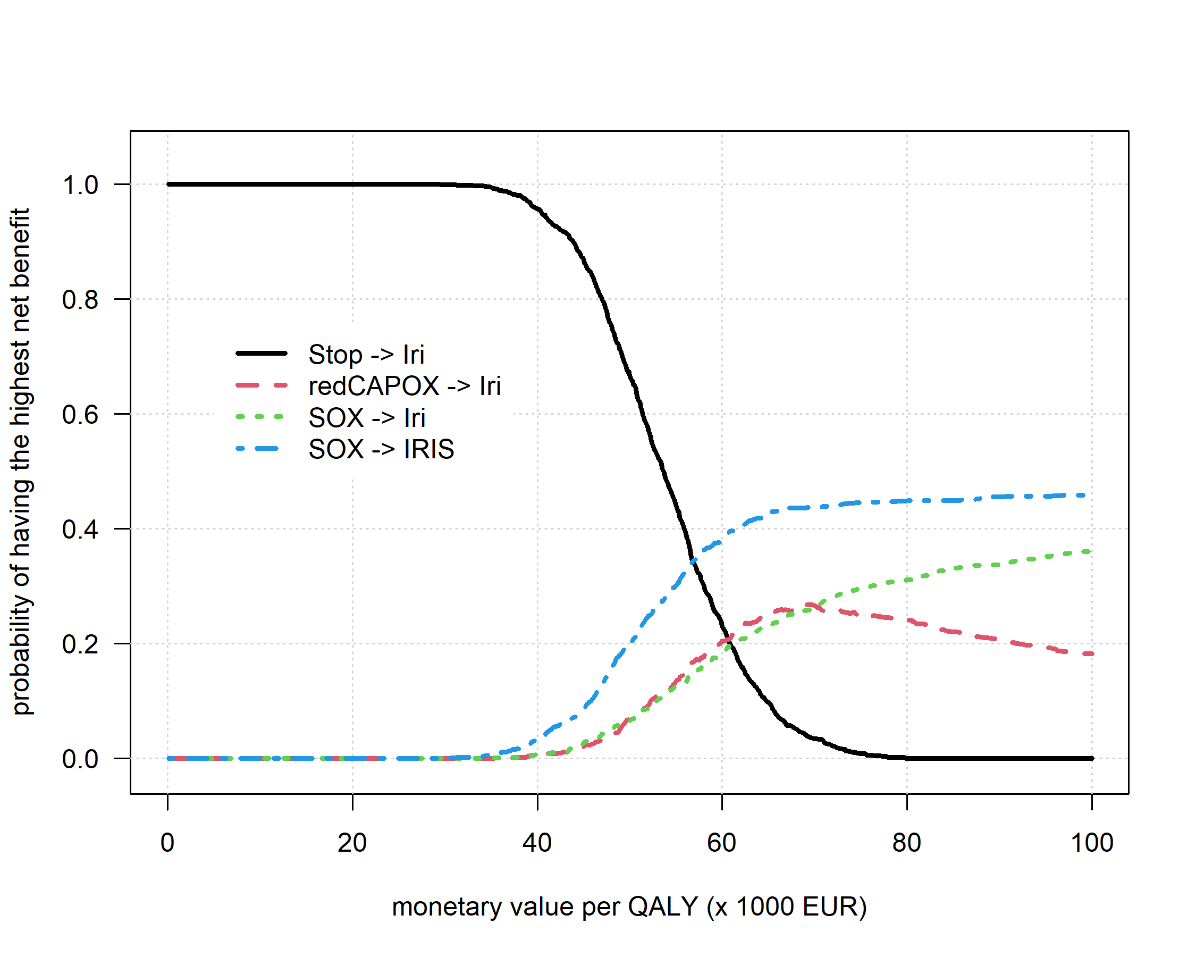
**Net benefit curve for the scenario where patients started with CAPOX.**


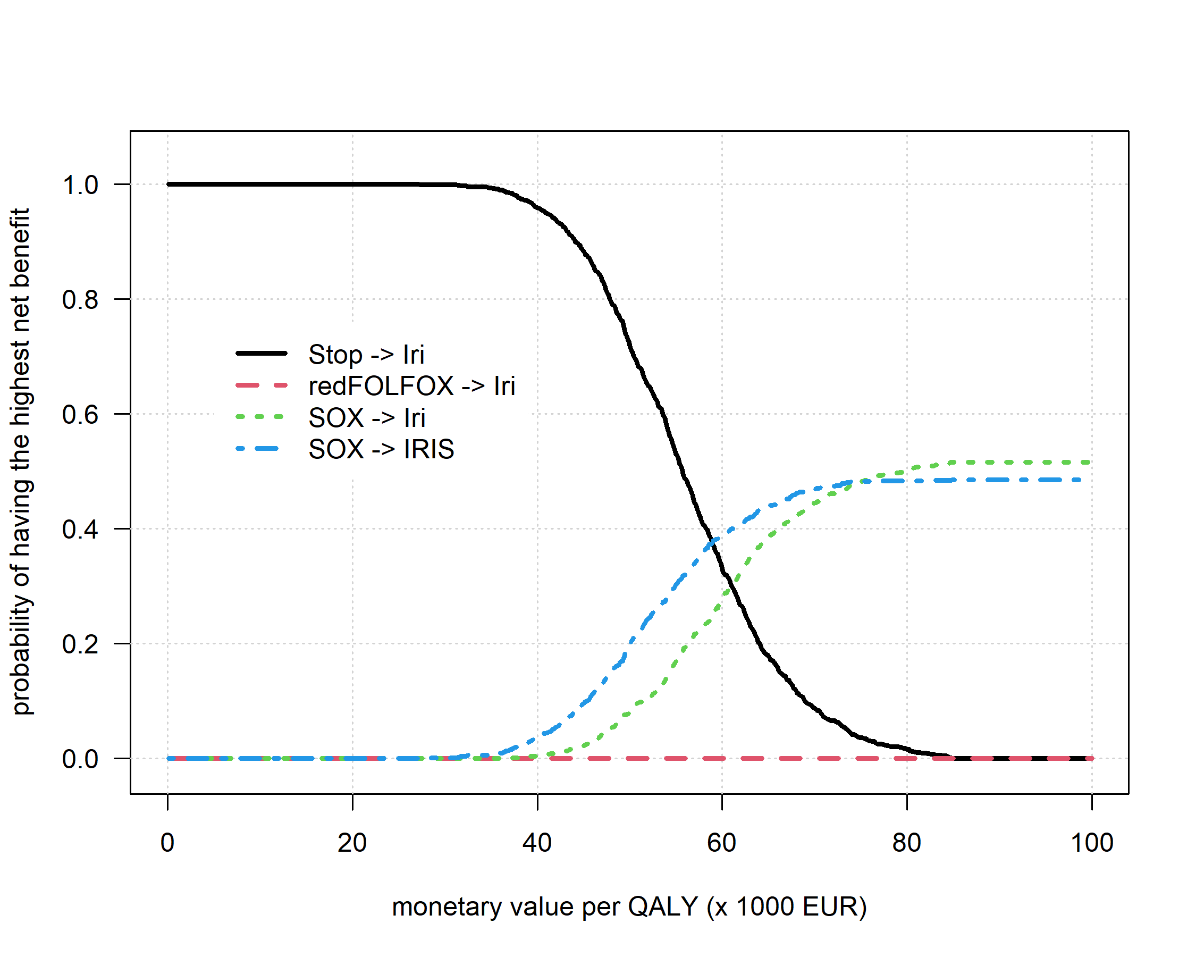
**B. Net benefit curve for the scenario where patients started with FOLFOX.**


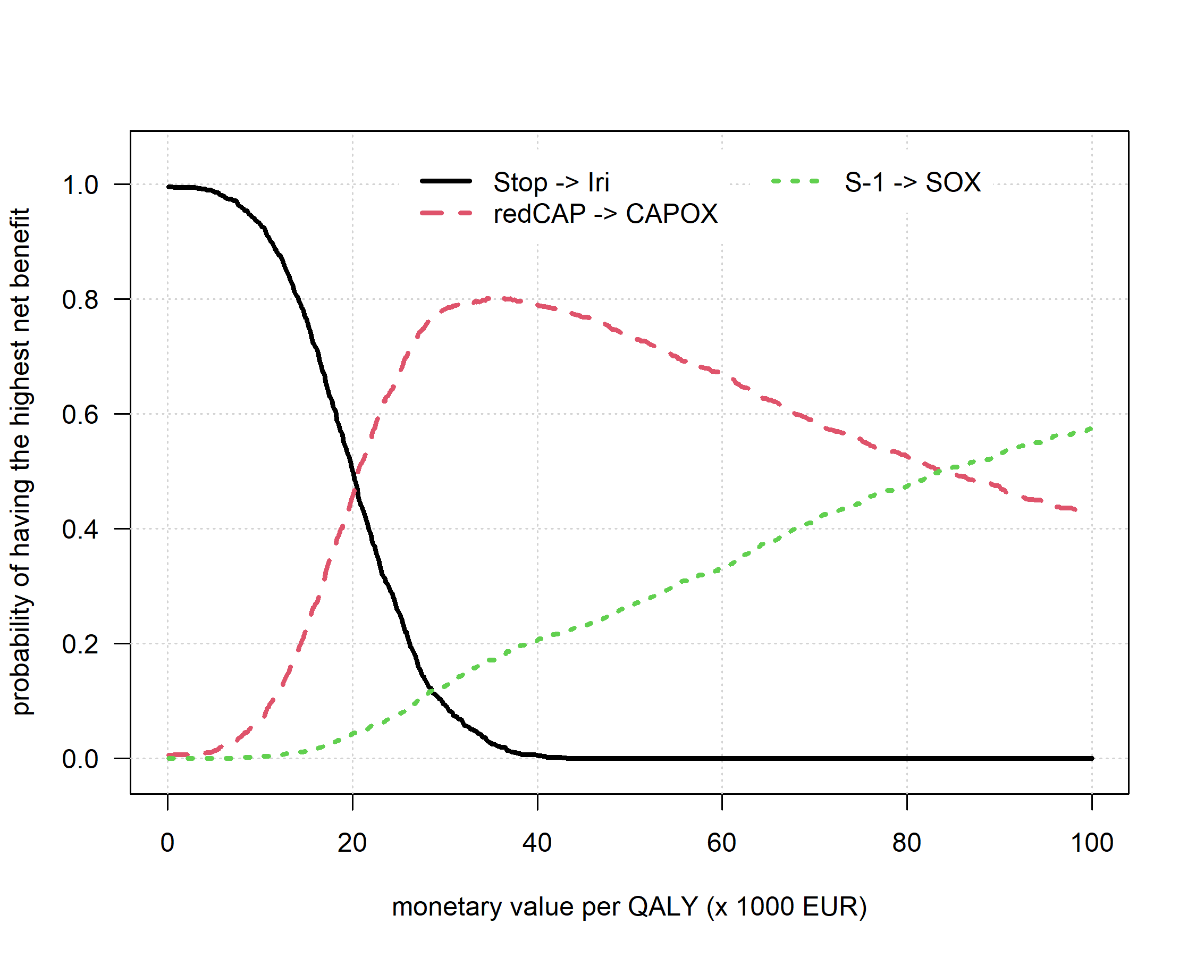
**C. Net benefit curve for the scenario where patients started with capecitabine.**
