# Supplementary material for: Cost-effectiveness of switching to S-1 after fluoropyrimidine-induced hand-foot syndrome or cardiovascular toxicity in the treatment of metastatic colorectal cancer
Source: ESMO Open. 2026 Mar 17;11(4):106304. doi: 10.1016/j.esmoop.2026.106304 (PMC13015576; doi:10.1016/j.esmoop.2026.106304)
Supplement: Supplementary Tables [file mmc2.docx]

**Supplementary Table Ia. Results from the sensitivity analysis with relative risk of 1.2 for no treatment (instead of 1.5).**

| **Strategy** | **Total costs per patient (€, EUR)** | **Costs**  **first-line**  **(€, EUR)** | **Costs second-line**  **(€, EUR)** | **Costs AEs (€, EUR)** | **Average QALYs**  **per patient** | **Median OS (months)** | **Median time-to-progression (months)** | **Average number of first-line treatment cycles** | **Average number of second-line treatment cycles** | **Percentage of patients with progression** | **ICER**  **(€ per 1 additional QALY)** |
| --- | --- | --- | --- | --- | --- | --- | --- | --- | --- | --- | --- |
| **Stop -> Irinotecan** | 8,074 | 0 | 8,074 | 0 | 0.93 | 12.9 | 11.5 | 0 | 8.1 | 63 | **Reference** |
| **reduced CAPOX**  **-> Irinotecan** | 22,112 | 12,842 | 9,163 | 107 | 1.04 | 14.5 | 12.8 | 12.1 | 8.9 | 64 | **122,454 (weakly dominated)** |
| **SOX -> Irinotecan** | 24,919 | 14,957 | 9,952 | 10 | 1.10 | 15.3 | 12.5 | 12.2 | 9.6 | 65 | **99,546** |
| **SOX -> IRIS** | 24,960 | 14,957 | 9,992 | 10 | 1.10 | 15.3 | 12.5 | 12.2 | 9.9 | 65 | **Equivalent** |

**Supplementary Table Ib. Results from the sensitivity analysis with relative risk of 1.2 for no treatment (instead of 1.5).**

| **Strategy** | **Total costs per patient (€, EUR)** | **Costs**  **first-line**  **(€, EUR)** | **Costs second-line**  **(€, EUR)** | **Costs AEs (€, EUR)** | **Average QALYs**  **per patient** | **Median OS (months)** | **Median time-to-progression (months)** | **Average number of first-line treatment cycles** | **Average number of second-line treatment cycles** | **Percentage of patients with progression** | **ICER**  **(€ per 1 additional QALY)** |
| --- | --- | --- | --- | --- | --- | --- | --- | --- | --- | --- | --- |
| **Stop -> Irinotecan** | 8,229 | 0 | 8,229 | 0 | 0.93 | 12.9 | 11.4 | 0 | 8.1 | 63 | **Reference** |
| **reduced FOLFOX**  **-> Irinotecan** | 46,569 | 37,299 | 9,163 | 107 | 1.04 | 14.5 | 12.8 | 18.3 | 8.9 | 64 | **Dominated** |
| **SOX -> Irinotecan** | 25,169 | 15,197 | 9,952 | 10 | 1.10 | 15.3 | 12.5 | 12.2 | 9.6 | 65 | **99,697** |
| **SOX -> IRIS** | 25,179 | 15,207 | 9,992 | 10 | 1.10 | 15.3 | 12.5 | 12.2 | 9.9 | 65 | **Equivalent** |

**Supplementary Table Ic. Results from the sensitivity analysis with relative risk of 1.2 for no treatment (instead of 1.5).**

| **Strategy** | **Total costs per patient (€, EUR)** | **Costs**  **first-line**  **(€, EUR)** | **Costs second-line**  **(€, EUR)** | **Costs AEs (€, EUR)** | **Average QALYs**  **per patient** | **Median OS (months)** | **Median time-to-progression (months)** | **Average number of first-line treatment cycles** | **Average number of second-line treatment cycles** | **Percentage of patients with progression** | **ICER**  **(€ per 1 additional QALY)** |
| --- | --- | --- | --- | --- | --- | --- | --- | --- | --- | --- | --- |
| **Stop -> Irinotecan** | 8,163 | 0 | 8,163 | 0 | 0.93 | 12.9 | 11.4 | 0 | 8.1 | 63 | **Reference** |
| **reduced CAP -> CAPOX** | 12,123 | 2,209 | 9,810 | 104 | 1.04 | 14.6 | 12.9 | 12.3 | 9.2 | 64 | **34,649** |
| **S-1 -> SOX** | 17,012 | 4,904 | 12,098 | 10 | 1.10 | 15.3 | 12.6 | 12.4 | 9.9 | 65 | **92,551** |

**ICER; incremental cost-effectiveness ratio, PSA; probabilistic sensitivity analysis**

**Supplementary Table IIa. Results from the sensitivity analysis with relative risk of 1.8 for no treatment (instead of 1.5).**

| **Strategy** | **Total costs per patient (€, EUR)** | **Costs**  **first-line**  **(€, EUR)** | **Costs second-line**  **(€, EUR)** | **Costs AEs (€, EUR)** | **Average QALYs**  **per patient** | **Median OS (months)** | **Median time-to-progression (months)** | **Average number of first-line treatment cycles** | **Average number of second-line treatment cycles** | **Percentage of patients with progression** | **ICER**  **(€ per 1 additional QALY)** |
| --- | --- | --- | --- | --- | --- | --- | --- | --- | --- | --- | --- |
| **Stop -> Irinotecan** | 6,079 | 0 | 6,097 | 0 | 0.71 | 9.8 | 8.9 | 0 | 6.0 | 63 | **Reference** |
| **reduced CAPOX**  **-> Irinotecan** | 22,112 | 12,842 | 9,163 | 107 | 1.04 | 14.5 | 12.8 | 12.1 | 8.9 | 64 | **47,666 (weakly dominated)** |
| **SOX -> Irinotecan** | 24,919 | 14,957 | 9,952 | 10 | 1.10 | 15.3 | 12.5 | 12.2 | 9.6 | 65 | **48,190** |
| **SOX -> IRIS** | 24,960 | 14,957 | 9,992 | 10 | 1.10 | 15.3 | 12.5 | 12.2 | 9.9 | 65 | **Equivalent** |

**Supplementary Table IIb. Results from the sensitivity analysis with relative risk of 1.8 for no treatment (instead of 1.5).**

| **Strategy** | **Total costs per patient (€, EUR)** | **Costs**  **first-line**  **(€, EUR)** | **Costs second-line**  **(€, EUR)** | **Costs AEs (€, EUR)** | **Average QALYs**  **per patient** | **Median OS (months)** | **Median time-to-progression (months)** | **Average number of first-line treatment cycles** | **Average number of second-line treatment cycles** | **Percentage of patients with progression** | **ICER**  **(€ per 1 additional QALY)** |
| --- | --- | --- | --- | --- | --- | --- | --- | --- | --- | --- | --- |
| **Stop -> Irinotecan** | 6,212 | 0 | 6,212 | 0 | 0.71 | 9.8 | 8.9 | 0 | 6.0 | 63 | **Reference** |
| **reduced FOLFOX**  **-> Irinotecan** | 46,569 | 37,299 | 9,163 | 107 | 1.04 | 14.5 | 12.8 | 18.3 | 8.9 | 64 | **Dominated** |
| **SOX -> Irinotecan** | 25,169 | 15,197 | 9,952 | 10 | 1.10 | 15.3 | 12.5 | 12.2 | 9.6 | 65 | **48,409** |
| **SOX -> IRIS** | 25,179 | 15,207 | 9,992 | 10 | 1.10 | 15.3 | 12.5 | 12.2 | 9.9 | 65 | **Equivalent** |

**Supplementary Table IIc. Results from the sensitivity analysis with relative risk of 1.8 for no treatment (instead of 1.5).**

| **Strategy** | **Total costs per patient (€, EUR)** | **Costs**  **first-line**  **(€, EUR)** | **Costs second-line**  **(€, EUR)** | **Costs AEs (€, EUR)** | **Average QALYs**  **per patient** | **Median OS (months)** | **Median time-to-progression (months)** | **Average number of first-line treatment cycles** | **Average number of second-line treatment cycles** | **Percentage of patients with progression** | **ICER**  **(€ per 1 additional QALY)** |
| --- | --- | --- | --- | --- | --- | --- | --- | --- | --- | --- | --- |
| **Stop -> Irinotecan** | 6,157 | 0 | 6,157 | 0 | 0.71 | 9.8 | 8.9 | 0 | 6.0 | 63 | **Reference** |
| **reduced CAP -> CAPOX** | 12,123 | 2,209 | 9,810 | 104 | 1.04 | 14.6 | 12.9 | 12.3 | 9.2 | 64 | **17,780** |
| **S-1 -> SOX** | 17,012 | 4,904 | 12,098 | 10 | 1.10 | 15.3 | 12.6 | 12.4 | 9.9 | 65 | **92,551** |

**ICER; incremental cost-effectiveness ratio, PSA; probabilistic sensitivity analysis**

**Supplementary Table IIIa. Results from the sensitivity analysis in which survival data from the SALTO trial was used from 9 weeks onwards (instead of from randomisation).**

| **Strategy** | **Total costs per patient (€, EUR)** | **Costs**  **first-line**  **(€, EUR)** | **Costs second-line**  **(€, EUR)** | **Costs AEs (€, EUR)** | **Average QALYs**  **per patient** | **Median OS (months)** | **Median time-to-progression (months)** | **Average number of first-line treatment cycles** | **Average number of second-line treatment cycles** | **Percentage of patients with progression** | **ICER**  **(€ per 1 additional QALY)** |
| --- | --- | --- | --- | --- | --- | --- | --- | --- | --- | --- | --- |
| **Stop -> Irinotecan** | 6,419 | 0 | 6,419 | 0 | 0.75 | 10.1 | 10.1 | 0 | 6.3 | 59 | **Reference** |
| **reduced CAPOX**  **-> Irinotecan** | 22,383 | 12,370 | 9,905 | 107 | 1.00 | 13.5 | 13.6 | 11.9 | 9.8 | 61 | **58,521 (weakly dominated)** |
| **SOX -> Irinotecan** | 24,193 | 14,440 | 9,753 | 10 | 1.07 | 14.3 | 13.4 | 11.8 | 9.6 | 62 | **56,647** |
| **SOX -> IRIS** | 24,233 | 14,440 | 9,793 | 10 | 1.07 | 14.3 | 13.4 | 11.8 | 9.6 | 62 | **Equivalent** |

**Supplementary Table IIIb. Results from the sensitivity analysis in which survival data from the SALTO trial was used from 9 weeks onwards (instead of from randomisation).**

| **Strategy** | **Total costs per patient (€, EUR)** | **Costs**  **first-line**  **(€, EUR)** | **Costs second-line**  **(€, EUR)** | **Costs AEs (€, EUR)** | **Average QALYs**  **per patient** | **Median OS (months)** | **Median time-to-progression (months)** | **Average number of first-line treatment cycles** | **Average number of second-line treatment cycles** | **Percentage of patients with progression** | **ICER**  **(€ per 1 additional QALY)** |
| --- | --- | --- | --- | --- | --- | --- | --- | --- | --- | --- | --- |
| **Stop -> Irinotecan** | 6,552 | 0 | 6,552 | 0 | 0.76 | 10.1 | 10.1 | 0 | 6.3 | 59 | **Reference** |
| **reduced FOLFOX**  **-> Irinotecan** | 44,871 | 35,704 | 9,905 | 107 | 1.01 | 13.6 | 13.6 | 17.4 | 9.2 | 61 | **Dominated** |
| **SOX -> Irinotecan** | 24,440 | 14,512 | 9,753 | 10 | 1.07 | 14.4 | 13.4 | 11.8 | 9.6 | 63 | **56,913** |
| **SOX -> IRIS** | 24,450 | 14,512 | 9,793 | 10 | 1.07 | 14.4 | 13.4 | 11.8 | 9.6 | 63 | **Equivalent** |

**Supplementary Table IIIc. Results from the sensitivity analysis in which survival data from the SALTO trial was used from 9 weeks onwards (instead of from randomisation).**

| **Strategy** | **Total costs per patient (€, EUR)** | **Costs**  **first-line**  **(€, EUR)** | **Costs second-line**  **(€, EUR)** | **Costs AEs (€, EUR)** | **Average QALYs**  **per patient** | **Median OS (months)** | **Median time-to-progression (months)** | **Average number of first-line treatment cycles** | **Average number of second-line treatment cycles** | **Percentage of patients with progression** | **ICER**  **(€ per 1 additional QALY)** |
| --- | --- | --- | --- | --- | --- | --- | --- | --- | --- | --- | --- |
| **Stop -> Irinotecan** | 6,495 | 0 | 6,495 | 0 | 0.75 | 10.1 | 10.1 | 0 | 6.3 | 59 | **Reference** |
| **reduced CAP -> CAPOX** | 11,762 | 2,124 | 9,638 | 104 | 1.01 | 13.6 | 14.0 | 11.8 | 9.0 | 61 | **20,662** |
| **S-1 -> SOX** | 16,581 | 4,734 | 11,847 | 10 | 1.07 | 14.3 | 13.5 | 11.9 | 9.7 | 62 | **85,721** |

**ICER; incremental cost-effectiveness ratio, PSA; probabilistic sensitivity analysis**
