# Supplementary material for: Cost-effectiveness of switching to S-1 after fluoropyrimidine-induced hand-foot syndrome or cardiovascular toxicity in the treatment of metastatic colorectal cancer
Source: ESMO Open. 2026 Mar 17;11(4):106304. doi: 10.1016/j.esmoop.2026.106304 (PMC13015576; doi:10.1016/j.esmoop.2026.106304)
Supplement: Supplementary Material 1 [file mmc3.docx]

**Supplementary material for those unfamiliar with cost-effectiveness analyses (CEAs) or health technology assessment (HTA)**

*Overview*

First, to take the step from clinical practice to decision modelling, we provided in **Methods** an overview of medication costs for a simplified treatment timeline for mCRC. We identified the most common treatment pathways where S-1 could be used as an alternative chemotherapy to 5FU or capecitabine and defined those as the treatment ‘strategies’ of interest for our study.

Next, we constructed a decision analytic model to aid in deciding between strategies by projecting their differences in terms of accumulated costs and effectiveness, which is expressed in terms of patients’ Quality-Adjusted Life Years (QALYs).^14^ We chose to use a cohort-level Markov model, its parameters informed by literature or data. The uncertainty in the model parameters was assessed using probabilistic sensitivity analysis (PSA), in which for every run, the parameters are drawn from their respective uncertainty distributions, running the model 5,000 times. We analysed the model runs to rank the strategies in terms of their average costs and QALYs, then determined the incremental cost per additional QALY. Finally, we used the net benefit framework to distinguish between cost-effective and ineffective strategies by expressing health gained in terms of monetary values and deducting costs.

To investigate the influence of certain parameters and decisions in the modelling process, we applied sensitivity analyses.

*Overall design of the Markov model*

The Markov model is a cohort-level model that follows a hypothetical cohort of 1,000 patients through their treatment trajectory.^14^ This model uses cycles of 1 week as time points to determine which ‘state’ patients are in at that time: either first-line treatment, progression, second-line treatment after progression, no second-line treatment after progression, and death. The state determines the medication that they are currently using, the associated costs and their QoL. Patients can move from one state to another, which is referred to as a transition. When or how often this occurs, is determined by transition probabilities. For instance, for a certain strategy, patients with HFS that occurred during CAPOX may continue CAPOX at reduced dose of capecitabine as their alternative first-line treatment. They may stay alive in that state for some time, accumulating costs and QALYs pertaining to that state, and then die, thereby transitioning to the state that represents death, after which they do not accumulate any more costs or QALYs.

*Uncertainty in the Markov model*

The model parameters are not known with absolute certainty; instead, they are estimates, and their uncertainty is important for correctly interpreting results. We incorporated the uncertainty in the parameters in our Markov model by running the model 1,000 times, and drawing model parameters from their uncertainty distributions. For instance, the relative risk (RR) of fluoropyrimidines versus S-1 was 0.93 in the systematic review, but the 99%CI reported by Derksen *et al*. was 0.81-1.07. This gives a standard error on the log scale of the RR of 0.058, which we used for our uncertainty distribution to repeatedly draw the RRs for the runs. This approach is called a probabilistic sensitivity analysis (PSA).^14^

*Example calculation*

We used the data from the SALTO trial to give an example of health and cost outcomes under a treatment strategy for the average patient, from the diagnosis of mCRC and starting first-line treatment to toxicity, progression, second-line treatment and finally death.^15^ This is to give readers who are unfamiliar with Markov modelling an idea of what the accumulated costs and QALYs look like.

Let us visualise the timeline and calculate the costs and QALYs that the typical patient would accumulate based on these survival data. Note that although our decision model starts after toxicity and the switch to alternative first-line treatment, for this didactical example we simply start at diagnosis of mCRC

See the **Figure.** We assume that after diagnosis, the patient starts with CAPOX as first-line treatment. After 3 cycles i.e. 9 weeks (costing €3,213) they experience HFS. They stop treatment, recover for 2 weeks, then continue CAPOX with a reduced dose for 9 cycles i.e. 27 weeks (costing €9,639) before progression. After progression, they take one week to meet with their clinician and decide what to do, and together they choose irinotecan monotherapy as second-line treatment, which they receive for an additional 9 cycles i.e. 27 weeks (costing €9,099) before their death. This patient’s total accumulated cost after 66 weeks (of which 63 in treatment) was €21,951. The patient lived for 38 weeks with a QoL of 0.83 (0.61 QALY), 1 week with a QoL of 0.73 (0.014 QALY) and 27 weeks with a QoL of 0.68 (0.35 QALY). 2 weeks of HFS with 0.15 disutility provide a subtraction of 0.006 QALY, giving a total rounded sum for the patient of 0.97 QALY that they accumulated. As an example calculation for net benefit: let us say that we value 1 QALY at €80,000. For the patient described above we thus ‘gained’ €80,000 times 0.97 i.e. €77,600 worth of ‘health effects’. But as we spent €21,951 on their treatment achieving this, our net benefit is €77,600 minus €21,951, yielding €55,649.

In our Markov model, the above calculations are carried out at the cohort level from toxicity onwards, for 1,000 patients that would undergo all the strategies as mentioned in **Methods**.


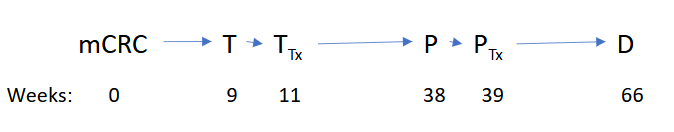
**Figure. Example timeline for the typical patient after diagnosis of mCRC.**

**mCRC: diagnosis of mCRC**

**T: toxicity**

**T_Tx_: reduced dose treatment after toxicity**

**P: progression**

**P_Tx_: second-line treatment after progression**

**D: death**
